# Supplementary material for: Never Change a Flowing System? The Effects of Retrograde Flow on Isolated Perfused Lungs and Vessels
Source: Cells. 2021 May 15;10(5):1210. doi: 10.3390/cells10051210 (PMC8156646; doi:10.3390/cells10051210)
Supplement: Supplementary file 1 [file cells-10-01210-s001.zip › cells-1187280-supplementary.pdf]

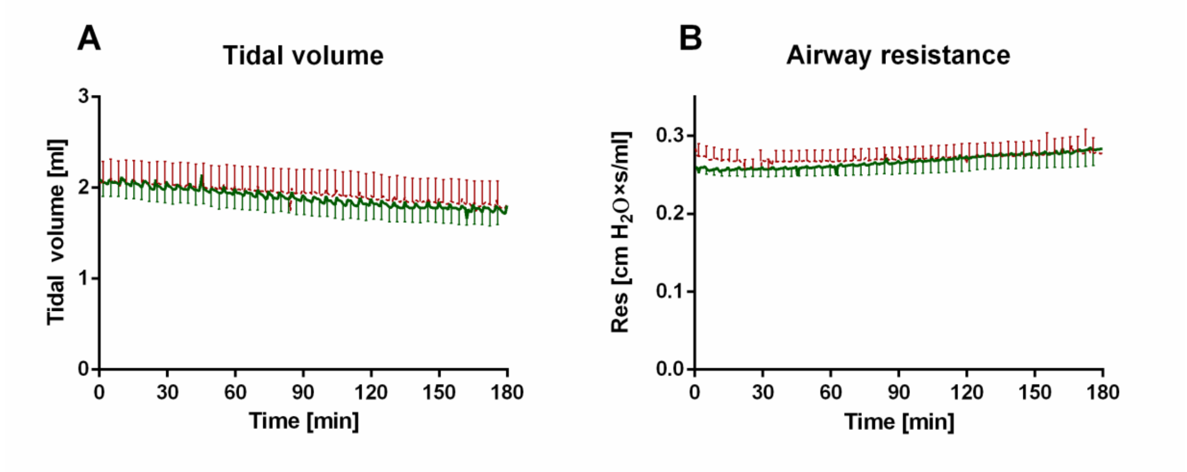

Figure S1. Influence of perfusion direction on physiological parameters in IPL. (**A**): Tidal volume (mean ± SEM), (**B**): Airway resistance (mean ± SEM). SEM is pruned to average of 5 min, *n* = 5.
